# Combat sports in virtual reality for rehabilitation and disability adaptation: a mini-review

**DOI:** 10.3389/fpubh.2025.1557338

**Published:** 2025-02-27

**Authors:** Yike Li, Chun Jiang, Hansen Li, Yuqin Su, Mengyao Li, Yang Cao, Guodong Zhang

**Affiliations:** ^1^Institute of Sport Science, College of Physical Education, Southwest University, Chongqing, China; ^2^Department of Police Tactics, Chongqing Police College, Chongqing, China; ^3^School of Physical Education, Sichuan Agricultural University, Ya'an, China; ^4^School of Physical Education, Shaanxi Normal University, Xi'an, China; ^5^Clinical Epidemiology and Biostatistics, School of Medical Sciences, Faculty of Medicine and Health, Orebro University, Orebro, Sweden; ^6^Unit of Integrative Epidemiology, Institute of Environmental Medicine, Karolinska Institutet, Stockholm, Sweden; ^7^International College, Krirk University, Bangkok, Thailand

**Keywords:** virtual reality, boxing, combat sports, rehabilitation, adaptive physical activities

## Abstract

This review examines the existing literature regarding the utilization of combat sports in virtual reality (VR) for disease rehabilitation and adaptive physical activity. A total of 18 studies were obtained from the Web of Science and Scopus databases. The results suggest that Boxing, the most studied combat sport in VR systems, has been primarily used to improve motor function and quality of life in patients with neurological conditions such as cerebral palsy, Parkinson’s disease, and stroke. Furthermore, VR combat sports have been shown to increase energy expenditure and physical activity intensity in individuals with disabilities, proving effective in maintaining overall physical health. Notably, VR boxing produces higher energy expenditure than other activities (e.g., tennis), with heart rate (HR) and oxygen consumption (VO2) during boxing sessions consistently exceeding those observed in tennis. Overall, research in this field remains limited and further explorations are warranted.

## 1 Introduction

Virtual Reality (VR) is a technology that uses computer simulations to create artificial realities, offering users a virtual environment that closely resembles the real world. Through devices like head-mounted displays (HMDs) (1) or 3D glasses, VR presents three-dimensional images to users, creating an immersive experience that allows real-time interaction with the virtual environment (2). Due to its affordability and lack of special requirements for use, VR has quickly gained popularity in fields such as education (3), sports (4), and entertainment (5), with increasing interest in its potential applications in rehabilitation and adaptive sports for individuals with disabilities.

Neurodegenerative and neurodevelopmental disorders, including Parkinson’s disease (PD), cerebral palsy (CP), and stroke, represent significant global health challenges due to their profound impact on motor function and quality of life. PD leads to motor impairments such as tremor, rigidity, bradykinesia, and postural instability, which severely hinder daily activities (6). CP results in abnormalities in muscle tone, movement, and motor skills, often leading to secondary complications such as hip pain, balance issues, and hand function impairment (7, 8). Stroke, a leading cause of adult disability, causes muscle weakness, spasticity, and cognitive deficits (9). For individuals affected by these conditions, even simple physical tasks, such as climbing stairs, can become considerable obstacles. These motor dysfunctions result in profound functional limitations and a marked decrease in life satisfaction (10, 11). As such, effective interventions for these disorders have garnered increasing attention.

In this regard, virtual reality (VR) has been utilized as a promising therapeutic approach to address these conditions, with exciting results in improving rehabilitation outcomes (12, 13). VR not only helps individuals with these disorders engage in tailored motor training but also offers a more engaging, interactive, and accessible form of rehabilitation. In addition, VR has demonstrated its effectiveness in reducing pain for injured athletes and enhancing their performance (14), while also providing cognitive skill training without the need for physical strain (15). Individuals with disabilities can also benefit from VR, such as experiencing effective training and rehabilitation without the limitations imposed by conventional therapies (16). Moreover, commercial VR game systems are gaining popularity in adaptive physical activity, with systems like Nintendo Wii Sports and Wii Fit offering immersive experiences that combine physical activity with engaging gameplay (17). These systems not only promote energy expenditure and physical activity but also contribute to maintaining overall health (18–20). Additionally, VR has been shown to provide high levels of enjoyment (21–23), potentially resulting in greater adherence to exercise routines (24).

Combat sports include many types, such as boxing, karate, taekwondo, wrestling, martial arts, judo, Muay Thai, and kickboxing. Combat sports are highly open and interactive activities, unconstrained by fixed routines or techniques, requiring athletes to continually adapt to new situations and respond quickly (25). Therefore, when integrating combat sports or related elements with electronic technologies or products, special attention must be given to their open and interactive nature. In this context, VR may be particularly well-suited for combining with combat sports or their elements, as VR is a technology that emphasizes proactivity and interactivity. Compared to other electronic media, VR increases users’ freedom of movement, allowing operators or players to fully immerse themselves and interact more conveniently and flexibly with others (such as real or virtual people) and their environment (such as virtual targets). These characteristics greatly enhance the enjoyment of such activities and may further increase participants’ physical activity levels, which can be particularly beneficial for those who need physical exercise to maintain basic health or recover from injury. Additionally, VR may overcome some of the limitations associated with the openness and interactivity of combat sports. Specifically, your training partner or opponent does not need to be physically present or even a real person. This is particularly useful for individuals in special settings, such as hospitals or rehabilitation centers.

To date, no study has specifically addressed the integration of combat sports with VR technology in academic efforts. Therefore, this mini-review is presented to explore the existing evidence regarding the effectiveness of incorporating combat sports into VR technology for disease treatment and adaptive sports among individuals with disabilities. By evaluating these studies, this paper seeks to provide insights and recommendations for future developments in this field.

## 2 Methods

We used keyword searches in WOS and Scopus (covering titles, abstracts, and keywords). Our search query was: Topic = “virtual reality” or “VR” AND Topic = “Boxing” OR “boxer” OR “combat sport*” OR “karate” OR “taekwondo” OR “wrestling” OR “fencing” OR “martial art*” OR “judo” OR “jiu jitsu” OR “wushu” OR “kung fu” OR “Muay Thai” OR “Krav Maga” OR “Sambo” OR “Aikido” OR “kickbox*”.

This search query was derived from a previous review on VR (26) and previous reviews on combat sports (27). We searched the databases from their inception to April 21, 2024. Exclusion criteria were as follows: (1) Unavailable full text; (2) Grey literature: For example, excluding conference papers, abstracts, theses, etc.; (3) Information unrelated to disease recovery or adaptive sports.

## 3 Results

We included 101 papers from WOS and 114 papers from Scopus. After removing 150 irrelevant and 47 duplicate documents, 18 papers were analyzed.

### 3.1 Potential of VR-based combat sports in rehabilitation and therapy

Virtual reality technology holds promise as a tool for therapy and improving rehabilitation outcomes (28–30). Often utilized in the form of virtual reality games, this technology aims to achieve its objectives by allowing patients to interact with various sensory environments. Nintendo Wii Sports is one of the most popular systems (31, 32). Wii games include activities such as tennis, baseball, boxing, and golf, providing patients with a variety of options. Based on the collected literature, boxing is the most common combat sport in this gaming system, primarily used for the treatment of individuals with cerebral palsy (CP), Parkinson’s disease (PD), and stroke.

Regarding cerebral palsy rehabilitation, the first published case report on using Wii for rehabilitation (33) documented the case of a 13-year-old CP patient who underwent 11 sessions of Wii Sports games, including boxing, lasting 60–90 min each (see Table 1). All games promoted trunk control, as boxing, for instance, requires trunk midline orientation and endurance of trunk muscles. Results showed positive outcomes in visual perception processing, postural control, and functional activities in CP patients using this gaming technology. Two studies explored the potential of using low-cost gaming systems as a therapeutic modality for CP patients, indicating the need for further research to ascertain its value (34, 35).

**Table 1:** Literature on VR-based combat sports in rehabilitation and therapy.

Paper	Disease/condition	Study type and design	Sample	Modality	During and frequency	Types of VR systems	Key outcomes
(33)	Cerebral Palsy	Retrospective and prospective case reports	A 13-year-old adolescent with spastic diplegic CP and delayed development	Boxing, Tennis, Bowling, Golf, Baseball	11 sessions over 4 weeks, each lasting 60 to 90 min	Wii sports (Wii remote and nunchuk)	The patient’s visual perceptual processing, postural control, and functional activities all improved significantly after rehabilitation with Wii sports.
(34)	Cerebral Palsy	Pre–post-test pilot study	6 children with dyskinetic CP, aged 6 to 12 years	Boxing, Tennis, Baseball	45 min each, twice a week for six weeks	Nintendo Wii (Wii Sports Resort disc and Wii Remote)	The Nintendo Wii could be a viable rehabilitation tool for children with CP in developing countries, with minor modifications allowing those with limited hand function or wheelchair dependence to participate.
(35)	Cerebral Palsy	Single-group experimental study	17 children with CP (mean age ± SD, 9.43 ± 1.51y)	Bowling, Tennis, Boxing, Dance	Familiarization 5 min; Gameplay 32 minutes; Rest 15 min	Active Video Games (Wii remote and nunchuk)	Active Video Games (such as boxing and dancing) can offer a moderate level of physical activity for children with cerebral palsy. They should be used selectively to achieve specific therapeutic goals.
(36)	Parkinson	Quasi-experiment pilot study	6 Parkinson’s patients	Tennis, Boxing	30 min each, three times a week for 6 weeks	Nintendo Wii	Wii sports can significantly improve patients’ range of motion, trunk mobility, balance, and quality of life. Patient satisfaction is extremely high.
(37)	Parkinson	Clinical trial	32 Parkinson’s patients (25 male, 71.50 ± 11.80 years)	Boxing	3 min each (Out of the 32 participants, 28 completed two training sessions)	HMD Oculus Quest 2; Elite strap and controllers	It is feasible and safe to use HMD equipment for boxing training in PD patients at different stages of the disease.
(38)	Stroke	Longitudinal follow-up study	5 hemiplegics patients (3 male, aged 18–60 years)	Tennis, Hula hoop games, soccer, boxing	60 min each, twice a week for 8 weeks	Nintendo Wii	After Nintendo Wii treatment, patients showed improvements in upper limb motor function and quality of life, which also persisted two months after treatment.
(39)	Stroke	Randomised, assessor-blinded clinical trial	15 patients with post-stroke duration of 135 ± 23 days (7 male, aged 40–60 years)	Table tennis, boxing, discus throwing games	45 min each, Five times a week for 4 weeks	Microsoft Kinect Xbox 360.	Virtual reality game therapy was more effective than neurodevelopmental treatment in improving upper limb motor recovery after stroke and equally effective in enhancing hand dexterity and trunk control.
(40)	Stroke	Randomized controlled clinical trial	20 hemiparetic stroke patients (aged 18–70 years)	Boxing	30 min/day, 3 sessions a week for 8 weeks	Xbox Kinect 360 game console	Both real boxing and virtual boxing are effective and safe in improving upper limb function, balance, and cognitive function in patients with hemiplegic stroke.
(41)	Pediatric Critical Care Unit (PCCU)	Prospective clinical trial	8 children in a PCCU (aged 4–16 years)	Boxing, Bowling	minimum of 10 min twice a day for 2 days	Nintendo Wii™	The patient’s upper limb activity during Wii™ was significantly greater than average daily activity and can be safely applied to a subset of critically ill children.
(42)	Pediatric intensive care unit (PICU)	Mini-ethnographic case study	2 male adolescents (15 and 13 years old) in the PICU	Movement to music, Boxing, Car racing, etc.	Case A: 4 times, average 18 min each; Case B: 2 times, average 35 min each	WalkinVR software; Oculus Rift HMD with 2 handled controllers	Adolescents with PICU who play VR games can achieve moderate levels of exercise intensity, which can be incorporated into early mobilization therapy for these patients.
(43)	Chronic low back pain (LBP)	Single case report	A 45-year old male with a 5-year history of disabling chronic low back pain	Boxing	Face-to-face: 3 times, 15 min each, Home: 6 times in a week, 15–25 min each.	Oculus Rift S HMD with connected Touch controllers, Oculus Quest	VR motion avatars are an interesting new way to help people with LBP overcome body image impairment and pain, and the benefits continued at three months of follow-up.
(44)	Post-polio syndrome (PPS)	Randomized, parallel, single-blind clinical trial	19 PPS patients (aged 40–75 years)	Tennis, Golf, Boxing, Bowling	50 min each, Twice a week for 7 weeks	Nintendo Wii	Interactive video games are safe and feasible for PPS patients, with significant improvements in dexterity, which continue to improve during follow-up.

In Parkinson’s disease rehabilitation, an experimental study involving six PD patients underwent 18 interventions, including boxing and tennis (Wii Sports), yielding satisfactory results. Nintendo Wii enhanced the range of motion, trunk activity, and balance in PD patients, thereby improving their quality of life (36). Another study investigated the feasibility of using commercial wearable head-mounted displays (HMDs) and selected immersive virtual reality (IVR) sports games (boxing exercise mode) for PD patients. Consistent with previous research, users highly rated IVR as a therapeutic tool and expressed willingness to recommend it to others. Moreover, the safety of wearable devices was further confirmed (37).

Regarding stroke rehabilitation, researchers noted that two months after intervention, Nintendo Wii continued to have a sustained positive impact on sensory-motor recovery and quality of life in stroke patients (38). Both neurodevelopmental treatment and virtual reality games can improve hand flexibility and trunk control in stroke patients, but virtual reality games play a greater role in enhancing upper limb movement recovery (39). Ersoy and Iyigun (40) compared virtual reality boxing training with real-world boxing training and found both to be effective in improving upper limb function, balance, and cognitive function in stroke patients, with no significant differences between them.

In addition to these three major conditions, VR gaming systems also play a role in the rehabilitation of other conditions. Specifically, Abdulsatar et al. (41) and Lai et al. (42) explored the feasibility of engaging critically ill children in physical activities in pediatric intensive care units, indicating that VR exercise can be safely implemented for some critically ill patients. During VR game activities (such as boxing), upper limb movement was significantly higher than the average daily activity level (*p* = 0.049). Additionally, gameplay appeared to enhance participants’ mood and alertness, and motivated them to engage more actively in early mobilization therapy. An intriguing study proposed a novel approach to treating chronic low back pain by embodying movement in VR (as a boxer), helping chronic low back pain patients make positive progress in body image (perceived strength, vulnerability, agility, and movement confidence) and pain (43). Finally, a total of 39 patients with Post-Polio Syndrome were randomly assigned to either the VR interactive game group (including boxing games) (*n* = 19) or the active exercise group (*n* = 20). Participants engaged in training twice a week (50 min per session) for a duration of seven weeks. Both groups showed improvements in motor function, functionality, balance, pain, and fatigue after the intervention; however, the VR game group demonstrated superior performance (44).

### 3.2 Role of VR-based combat sports in adaptive physical activities

VR plays essential roles in adaptive physical activities (16, 45). For individuals with disabilities, participating in regular sports activities may be unattainable, and a lack of activity could further deteriorate their health condition. Therefore, the advantages of virtual reality become pronounced in such scenarios.

Firstly, VR-based combat sports help increase energy expenditure in individuals with disabilities (see Table 2). An experiment tested the energy expenditure of 10 chronic stroke patients using Wii Sports for 15 min each of boxing and tennis activities (20). Due to physical limitations, 2 subjects could not complete the boxing game, and 3 did not participate in the tennis game. Except for one tennis participant, all other participants had an energy expenditure of ≥3 MET during the game sessions, with boxing (4.1 MET) slightly higher than tennis (3.7 MET), but the difference was not significant. The perceived exertion during boxing (5.3 MET) was higher than tennis (4.1 MET) (*p* < 0.05). Wii boxing, whether in single-player or multiplayer mode, provided moderate-level (MET >3.3) physical activity opportunities for children with unilateral cerebral palsy (19). The frequency of punching with the non-dominant arm was significantly higher in single-player mode, possibly because children are more relaxed without competition. In multiplayer mode, the frequency of punching with the dominant arm was higher, though not significant. Participants reported a preference for multiplayer games, and that the interactivity and enjoyable nature of multiplayer games may drive them to use Wii sports more actively. However, compared with single-player games, this may not be conducive to the recovery and treatment of their hemiplegic hand.

**Table 2:** Literature related to VR in adapted sports.

Article	Country	Participants	N (sex)	Types of VR systems	Sports	During	Test index	Results
(20)	Netherlands	Chronic (≥ 6 months) stroke patients	10 (no specific)	The Nintendo Wii the Wii remote (wireless) the Nunchuk	Tennis and boxing	30 min	Oxygen uptake heart rate (HR) Fat free mass Pulmonary ventilation Respiratory exchange rate Metabolic equivalent (MET) perceived exertion	The mean energy expenditure during Wii Sports game play was 3.7 (± 0.6) MET for tennis and 4.1 (± 0.7) MET for boxing
(19)	Canada	Hemiplegic cerebral palsy	15 (10 M, 5F)	“Wii Sports” package with the Wii Remote and Nunchuck (Nintendo, Inc.).	boxing	16 min	Oxygen consumption (VO_2_) MET energy expenditure HR perceived xertion	Moderate levels of physical activity were obtained during both multiplayer and solo play with no significant differences in energy levels or HR observed
(46)	Brazil	Chronic hemiparetic stroke	12 (10 M, 2F)	console Xbox 360 + Kinect	tennis and boxing	38 min	HR VO_2_ Anaerobic Threshold (AT) Respiratory Compensation Point (RCP)	Heart rate during virtual reality games was similar to AT and significantly lower than RCP (*p* ≤ 0.05), while VO_2_ was significantly lower than AT and RCP (*p* < 0.05).
(47)	Brazil	Paraplegic individuals with spinal cord injury	8 (all men)	Xbox360+ Kynect^®^ console^e^	tennis and boxing	41 min	HR VO_2_ AT RCP	Mean energy expenditure during the active video games sessions was 2.4 METS, characterizing a low-intensity aerobic exercise.
(48)	Norway	Spinal cord injuries who rely on wheelchairs	24 (22 M, 2F)	X-box Kinect Nintendo Wii VR Oculus Rift	boxing	45 min	peak oxygen uptake Peak heart rate	Approximately 30 of the 45 min of exergaming was performed at moderate or high intensity.
(49)	Poland	Wheelchair boxers with different types of disabilities	11 (all men)	Oculus Quest 2 wireless VR headset VR glasses and controllers	boxing	30 min	Percentage of maximal heart rate (%HRmax) Average exercise heart rate (HRave) Perceived exertion Physical Activity Enjoyment Scale	The exercise intensity of the athletes during wheelchair boxing training in VR is at a beneficial moderate level for health (HRave = 68.98% HRmax)

Secondly, VR-based combat sports help increase the intensity of physical activity. Regarding the evaluation of aerobic exercise intensity using the anaerobic threshold (AT) and respiratory compensation point (RCP), researchers organized four interventions consisting of virtual reality games (VRG) for 12 chronic hemiparetic stroke survivors, with 3 min of tennis, 1-min match change, and 4 min of boxing per group, with a 2-min interval between groups (46). Such interventions did not provide sufficient aerobic stimulus. Although heart rate (HR) and oxygen consumption (VO_2_) showed good reproducibility during VRG, VO_2_ was significantly lower than the AT and RCP, indicating only low-intensity aerobic exercise levels. Therefore, to enhance the aerobic capacity of stroke survivors through VRG, it is necessary to increase the intensity or volume of training. Additionally, authors found that HR and VO_2_ during boxing matches were always higher than tennis matches. A similar study further validated these findings in 8 spinal cord injury paraplegic patients (47). None reached the AT level during tennis matches, but 5 did during boxing matches, and energy expenditure (EE) during boxing matches of 3.0 (2.0–3.9). The MET values were also higher than those in tennis matches (mean = 2.3). However, Three-quarters of the participants had an EE below 3 MET during the entire game movement period.

Considering the physical limitations of individuals with disabilities, using relative exercise intensity (i.e., as a percentage of peak oxygen consumption or peak heart rate) is also a reasonable option. In a study, 24 wheelchair-dependent spinal cord injury patients played 3 different sports games (X-box Kinect, Fruit Ninja; Nintendo Wii, Wii Sports Boxing; VR Oculus Rift, boxing) for 15 min each with a 5-min break in between (48). Participants exercised an average of 6.6 min at high intensity, 24.5 min at moderate intensity, and 13.9 min at low intensity during the games (48). There was no significant difference in the time spent at moderate exercise intensity among the three sports games. Interestingly, participants engaged in significantly more high-intensity exercise during VR compared to Kinect.

Another study assessed the exercise intensity level of 11 wheelchair boxers during VR training using the average percentage of maximum heart rate (49). This study found that virtual reality games were highly effective in maintaining the physical health of individuals with disabilities, achieving a moderate health level. Users also rated VR games very highly. Additionally, the authors found that an additional 0.5 kg of hand-held weight did not significantly change the heart rate and intensity during training (49).

## 4 Discussion and future directions

We found that there were relatively few literature on VR technology in combat sports, with the majority of interest focused on using virtual reality games for patient therapy, providing them with physical activity and energy expenditure. This further underscores the critical role of integrating VR with combat sports in advancing this field.

Regarding the research on the energy expenditure of virtual reality games for individuals with disabilities, tennis and boxing are two popular sports. Three papers indicated that energy expenditure during VR boxing was higher than during tennis, including heart rate and VO_2_ (20, 46, 47). Two studies demonstrated that VR games could achieve a MET level of 3 for users (19, 20), while another study reported the opposite result (47), with 75% of individuals having energy expenditure below 3 MET during the game. This discrepancy may be related to different types of disabilities among users and inconsistent durations of gameplay. Therefore, future research should investigate the moderating effects by disabilities and focus on the effective duration. Existing literature indicates that VR games can only achieve low-intensity aerobic exercise standards for patients (46, 47). Subsequent research should explore ways to increase aerobic exercise intensity to better meet the exercise needs of individuals with disabilities.

Currently, we have observed that among various combat sports, only boxing has been integrated with VR technology for medical applications or adaptive sports for individuals with disabilities. Given the proactive nature of combat sports and their emphasis on dynamic interaction with others and the environment, it raises the question: could incorporating other sports, such as taekwondo or kickboxing, lead to comparable results? These sports, which involve significant lower-body engagement, may potentially increase energy expenditure in individuals with disabilities, addressing the challenge of insufficient energy consumption. Exploring these possibilities represents an important direction for future research. Moreover, the inclusion of such activities could provide patients with the opportunity to enjoy diverse forms of exercise, fostering their motivation to engage consistently in rehabilitation programs or more energy-intensive activities.

## 5 Conclusion

From the unique perspective of combat sports, we reviewed the role of VR technology in healthcare and adaptive sports for individuals with disabilities. Virtual reality games have demonstrated significant value by addressing challenges that traditional therapy and training methods cannot fully overcome. However, exploration in this area remains insufficient. As a nascent field, it presents substantial opportunities for further investigation and innovation. In conclusion, our findings highlight that the integration of VR technology into combat sports represents a highly promising domain with tremendous potential for future development.
